# Verification of the Propagation Range of Respiratory Strain Using Signal Waveform Measured by FBG Sensors

**DOI:** 10.3390/s20247076

**Published:** 2020-12-10

**Authors:** Shouhei Koyama, Atsushi Fujimoto, Yuma Yasuda, Yuuki Satou

**Affiliations:** 1 Faculty of Textile Science and Technology, Shinshu University, 3-15-1 Tokida, Ueda City, Nagano 390-8621, Japan; 17f1027d@shinshu-u.ac.jp; 2 Institute for Fiber Engineering, Shinshu University, 3-15-1 Tokida, Ueda City, Nagano 390-8621, Japan; 3 Graduate School of Science and Technology, Shinshu University, 3-15-1 Tokida, Ueda City, Nagano 390-8621, Japan; 19fs118j@shinshu-u.ac.jp (A.F.); 20fs120f@shinshu-u.ac.jp (Y.Y.)

**Keywords:** fiber Bragg grating sensor, respiratory strain, breathing, propagation range, signal waveform, vital signs

## Abstract

The FBG (Fiber Bragg grating) sensor is an optical fiber type strain sensor. When a person breathes, strain occurs in the lungs and diaphragm. This was verified using an FBG sensor to which part of the living body this respiratory strain propagates. When measured in the abdomen, the signal waveforms were significantly different between breathing and apnea. The respiratory cycle measured by the temperature sensor attached to the mask and the strain cycle measured by the FBG sensor almost matched. Respiratory strain was measured in the abdomen, chest, and shoulder, and the signal amplitude decreased with distance from the abdomen. In addition, the respiratory rate could be calculated from the measured strain signal. On the other hand, respiratory strain did not propagate to the elbows and wrists, which were off the trunk, and the respiratory time, based on the signal period, could not be calculated at these parts. Therefore, it was shown that respiratory strain propagated in the trunk from the abdomen to the shoulder, but not in the peripheral parts of the elbow and wrist.

## 1. Introduction

**Figure 1:**
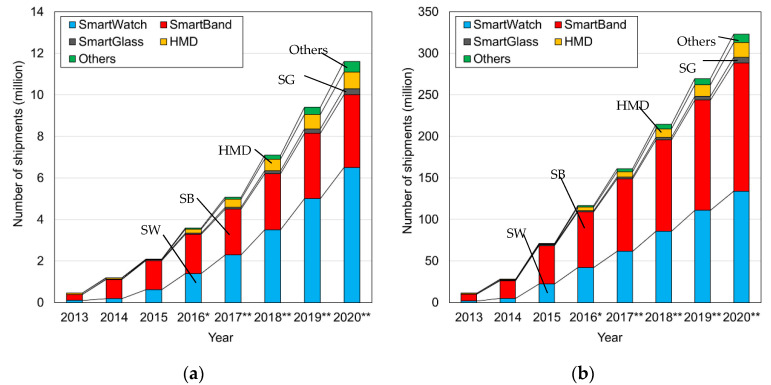
Market size and forecast of wearable devices for each year, (Source) Yano Research Institute Ltd., “Global Wearable Device Market: Key Research Findings 2016”, (**a**) Market size in Japan, (**b**) Global market size. * The value of market size figures in 2016 were expected. ** Market size figures after 2017 were forecasts. (HMD: Head-Mount Displays) [7].

A vital sign is a measurable biometric signal produced by a living body that indicates life, and such signals generally pertain to variables of pulse rate, respiration, body temperature, and blood pressure [1,2,3]. In order to maintain life, humans take in oxygen in the lungs, and the contraction of the heart sends oxygen to cells throughout the body by blood. Heat and blood flow pressure are generated by the movement of these organs, and biological information can be grasped from these four vital signs. Paramedics and nurses routinely measure these vital signs of patients, from which they detect valuable health-status information. Opportunities to measure vital signs in daily life are often measured after an abnormality occurs in the physical condition, such as “instructed by a doctor” or “feeling abnormal in physical condition”. Even when instructed by a doctor, vitals often measured regularly at fixed times, such as when waking up or before and after meals, and it is not common to measure continuously on a regular basis. There are many countries where the elderly population is on the rise, with Japan at the top. In recent years, manufacturers have developed wearable vital sensors that can automatically measure biological information in daily life to maintain the health of the elderly [4,5,6]. The wearable sensor has the advantage that it can be attached to a living body, so it can measure vital signs anytime, anywhere. Therefore, the user can constantly measure vital signs while living a daily life just by wearing a sensor. As a result, detailed information, such as the transition of vital signs in daily life, can be measured, so that one can know more about one’s physical condition and can receive a diagnosis based on more information from a doctor. Figure 1 shows the market size forecast for wearable devices released by the Ministry of Internal Affairs and Communications of Japan [7]. From Figure 1, it can be seen that the market size is expected to expand both in Japan and around the world, and that it will grow by about four times in the five years from 2015. By these projections, the wearable sensor industry is growing. With wearable sensor products, the pulse rate is mainly measured using a photoelectric pulse wave sensor. The principle of this sensor is Photoplethysmography (PPG), which measures light absorption phenomenon by hemoglobin by inserting light into the living body [8,9]. This sensor uses a green LED (about 550 nm) with a high absorption coefficient of hemoglobin oxide in blood vessels as a light source. The green light inserted into the living body from the light source is absorbed by the oxidized hemoglobin inside the capillaries and reflected to the outside of the living body. This reflected light is detected by a detector. The amount of oxidized hemoglobin in the capillaries changes as the heart contracts, and the signal intensity corresponding to that amount is detected. The measured signal intensity change is related to the contraction of the heart, and the heartbeat cycle can be calculated by detecting the peak intensity of the measured signal and can be calculated as the pulse rate. When the heart contracts, the amount of oxidized hemoglobin is the largest, so the measured output signal power value has the smallest. Because green light has a short penetration depth into living organisms, transmission-type sensors that use infrared light that penetrates deeper have also been developed. Such products can be used to detect early signs of stress problems and heat stroke. However, the photoelectric pulse wave sensor cannot measure respiratory rate and blood pressure, which are indicators of vital signs, at the same time.

On the other hand, as a sensor for measuring the respiratory rate, a temperature sensor using a thermistor and a pressure sensor installed on the nose are common [10,11,12,13]. Breathing is performed by up/down changes in the diaphragm and expansion/contraction changes in the lungs. Studies have measured the strain caused by breathing using FBG (Fiber Bragg grating) sensors and have calculated respiratory rates based on these. Such a method was installed in a bed/cushion [14,15,16] an FBG sensor embedded in a belt-like cloth wound around the chest [17,18,19]. Massaroni et al. measured the strain caused by the expansion and contraction of the lungs using an FBG sensor installed on the upper human body and calculated parameters related to respiration [20,21]. Thus, extant research has produced devices for measuring more real-time and complex respiratory parameters using FBG sensors. However, these studies only measure respiratory rate and cannot measure multiple vital signs at the same time. Furthermore, these studies have not well-verified which part of the body the strain by the lung and the diaphragm expansion and contraction propagates.

To interpret measured biological information in detail, it is useful to measure and assess multiple vital signs simultaneously [22,23,24]. Thus, more complex wearable sensors are highly sought for measuring pulse rate, respiratory rate, and blood pressure at all times. Our major research proposal is to develop a wearable multi-vital-sign sensor. For this purpose, an optical fiber Bragg grating (FBG) sensor is used. In our previous work, an FBG sensor was installed at the pulsation point of the wrist strain signal so that pulsation could be measured. From the measured signals (e.g., pulse rate [25], respiratory rate [26], blood pressure [27,28], and blood glucose value [29]), the biological parameters of the circulatory system can be assessed in real time. The pulmonary rate was calculated by measuring respiratory sinus arrhythmia, which reflects changes in pulse rate caused by changes in the autonomic nervous system. To calculate the respiratory rate using this method, the pulsation strain must be measured in a stationary state for approximately 30 s. Unfortunately, this particular capability lacks real-time calculation. To calculate the respiratory rate, the strain caused by respiration emerging from the living body must be directly measured in real time.

We propose a method using two FBG sensors to develop a sensor that simultaneously measures pulse rate, blood pressure, and respiratory rate. The important point of this method is the installation part of each FBG sensor. One FBG sensor is installed at the pulsation point on the wrist or elbow to calculate pulse rate and blood pressure. The other FBG sensor is used to measure respiratory rate. This FBG sensor should be installed in a part where respiratory strain by movement of the lungs and diaphragm can be detected. For that purpose, it is necessary to confirm to what part of the living body these strains propagate. The part where these strains can be detected most is the optimum installation part for the FBG sensor. Furthermore, the effect of respiratory strain on the pulsation points of the elbows and wrists has not been well-clarified. At the pulsation point where the strain propagated from the heart is detected, the strain due to respiration becomes noise. On the other hand, when only respiratory strain is detected, cardiac strain becomes noise. Therefore, it is necessary to confirm whether the strain due to respiration propagates to the pulsation point in the living body. In this study, we verify the extent to which the strain from a living body caused by respiration propagates. There are five measurement areas for this purpose: abdomen, chest, shoulder, elbow, and wrist. We verify that the strain signal caused by respiration can be detected from signals measured at each location. Additionally, a measurement method for simultaneously calculating multiple vital signs is considered.

## 2. Materials and Methods

### 2.1. FBG Sensor Measurement Principle and Measurement System

The FBG sensor is a PANDA-type polarization-maintaining single-mode optical fiber (SM15-PS-U25D, Fujikura Co., Ltd., Tokyo, Japan.) made of silica glass. In the sensor part, a diffraction grating, having an organic refractive index change, is installed at the core part of the optical fiber. When the FBG sensor was fabricated, the optical fiber protection films—at the location at which the FBG sensor will be engraved by using the phase mask—were removed. After the FBG sensor was engraved with a phase mask, this part was recoated with UV curable resin (UV acrylate), which is the same material as the resin stripped from the optical fiber. The FBG sensor was used in its fiber optic shape. When near-infrared light reaches this sensor, only a specific wavelength is reflected according to Equation (1):

(1) $$\lambda_{Bragg}=2n_{eff}\Lambda$$

where *λ_Bragg_* is the Bragg wavelength, *n_eff_* is the refractive index of the optical fiber core, and Λ is the diffraction grating spacing. This reflected wavelength is called the “Bragg wavelength,” and it is detected by the FBG sensor system. When strain is applied to the sensor, the optical fiber is stretched. Therefore, the diffraction grating spacing (i.e., the FBG sensor) also becomes longer. According to Equation (1), the larger the grating spacing, Λ, the larger the Bragg wavelength, *λ_Bragg_*. Thus, a wavelength longer than the base Bragg wavelength in the undistorted state is detected. The measured signal from the FBG sensor shows the shift length from the base Bragg wavelength on the vertical axis. Therefore, on the vertical axis, “0” indicates that no strain is applied, “+side” indicates that the optical fiber is stretched because of strain, and “−side” indicates that the optical fiber is contracted. It also shows that the larger the absolute value on the vertical axis, the larger the stretch. During inspiration, the abdomen and chest expand due to the movement of the diaphragm and lungs. When FBG sensors are installed in those areas, the optical fiber is stretched, which increases the spacing between the diffraction gratings. The signal of the FBG sensor is measured on the “+side” in the vertical axis direction. On the other hand, during exhalation, the abdomen and chest contract. Since the FBG sensors installed in those areas also shrink, the spacing between the diffraction gratings becomes smaller. Therefore, the signal of the FBG sensor is measured on the “−side” in the vertical axis direction. In this way, the expansion and contraction of the living body by respiration can be detected by the change in the waveform of the measurement signal due to the expansion and contraction of the FBG sensor.

**Figure 2:**
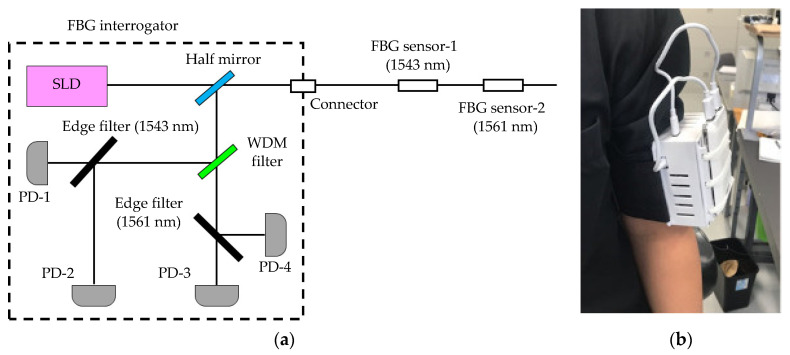
Diagram and photo of FBG sensor system. (**a**) FBG interrogator and FBG sensor; (**b**) developed edge-filter module-type FBG interrogator.

This system comprises an FBG interrogator in which the light-source and detection units are packaged and an optical fiber in which the FBG sensor is installed. The outline of the FBG interrogator is shown in Figure 2. Our edge-filter module is used with the FBG interrogator [30]. This edge filter has polarization dependent loss. This polarization-dependent loss had a large effect on the measured signal waveform; therefore, this loss had to be reduced. The light source SLD emits polarized light. Bragg wavelength light had to be reached at the edge filter while maintaining this polarized light, so PANDA-type polarization-maintaining single-mode optical fiber was used. In this FBG interrogator, when the light of the Bragg wavelength reached the edge filter, it was divided into reflected light and transmitted light. The amount of divided reflected light and transmitted light was detected by each photodiode. The detected light amount was calculated by (R − T)/(R + T) from the reflected light amount R and the transmitted light amount T. There was nothing in the strain (base Bragg wavelength); the amount of reflected light and transmitted light were each divided by 50%, and the output from the FBG interrogator was 0. When the FBG sensor was extended, the Bragg wavelength became longer, the amount of reflected light from the edge filter increased, and the amount of transmitted light decreased, so the output became “+”. Conversely, when the FBG sensor contracted, the Bragg wavelength became shorter, and the output of the FBG interrogator became “−”. 

Two FBG sensors, with different base Bragg wavelengths, were installed in one optical fiber. The near-infrared light emitted from the light source was inserted into the core of the optical fiber and reached each sensor. The FBG sensor-1 reflected near-infrared light in the 1543 nm band, and the FBG sensor-2 reflected its Bragg wavelength in the 1561 nm band. Each near-infrared light signal was detected, and the displacement length of each Bragg wavelength from the time of calibration was calculated. The calibration was performed using the FBG sensor installed on the desk and without the strain. The sampling rate of the FBG sensor was 1 kHz. The measured signal was processed by a 0.03 to 0.5 Hz bandpass filter (BPF) to remove noise. Therefore, the BPF-processed signal (vertical axis: output value from the BPF-processed FBG sensor, horizontal axis: measurement time) was used as the signal measured by the FBG sensor.

### 2.2. Simultaneous Measurement Experiment during Breathing Using FBG Sensor and Respiratory Temperature Sensor

**Figure 3:**
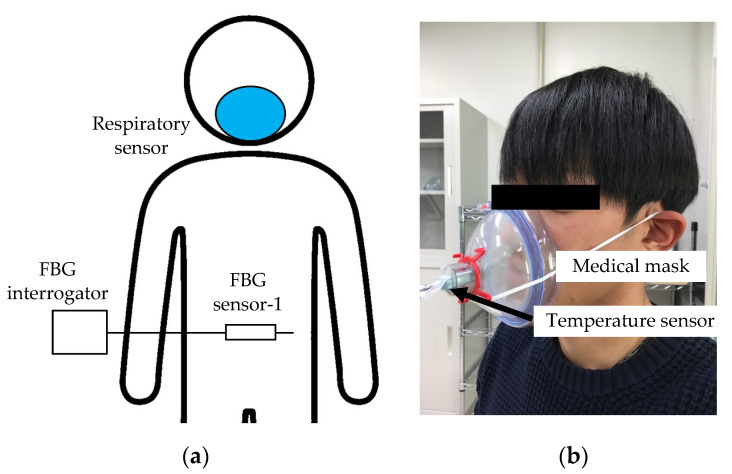
Schematic of verification experiment of measurement signal of FBG sensor and respiration time by respiratory sensor, and photograph of respiration sensor. (**a**) Measurement points of FBG sensor in the experiment; (**b**) Respiratory sensor with temperature sensor and medical mask.

The relationship between the FBG sensor measurement signal and the respiration time was also verified. As shown in Figure 3, a medical mask was attached to the subject’s oral cavity and an FBG sensor was installed on the abdomen. A thermistor type temperature sensor (Sato Keiryoki MFG. Co., Ltd., Tokyo, Japan. SK-L200TH2α) was installed in the airflow path of the medical mask. This temperature sensor measured the outside air temperature during inspiration and the exhaled air temperature from inside the body during expiration. Because each temperature was different, the temperatures measured during inspiration and expiration differed. Therefore, because the temperature changes periodically during inspiration and expiration, one cycle of the measurement signal was calculated as one respiration time. The sampling frequency of this temperature sensor was 1 Hz, and the temperature resolution was 0.1 °C. Each sensor was measured simultaneously when the subject breathed. The time synchronization of both sensors was performed by the following method. First, each sensor was installed to the subject, and the measurement start button of each sensor was clicked at the same time. The subject stopped breathing for 5 s from the start of measurement, and then started breathing. The fluctuation point of the signal measured after 5 s was treated as the measurement start point (Measurement time is 0), and the measurement signals at each sensor were synchronized in time. The time for one respiration was 6 s (inspiration: 3 s, expiration: 3 s). The measurement time was approximately 60 s and approximately 10 respirations were measured in one measurement; four measurements were taken. Because the signal measured by the FBG sensor also changed periodically, the time of each cycle was calculated. The time calculated by each sensor was compared and the respiration time from the signal measured by the FBG sensor was verified.

The FBG sensor was very sensitive to temperature, so it was necessary to pay attention to the temperature crossing sensitivity. The following measurement environment adjustment and calibration measurements were performed in advance in all experiments. The room temperature and humidity were unified at 20 degrees and 65% RH. Immediately after the FBG sensor was installed to the living body, the Bragg wavelength was shifted due to the temperature difference. However, it was confirmed that the baseline fluctuation of the measurement signal did not appear as a calibration measurement 30 min after the FBG sensor was installed to the living body. The temperature of the living body did not change rapidly during the subsequent signal measurement. By these countermeasures, the measurement signal had almost no effect on the temperature cross-sensitivity.

### 2.3. Measurement Experiment of Respiratory Strain Using FBG Sensor

**Figure 4:**
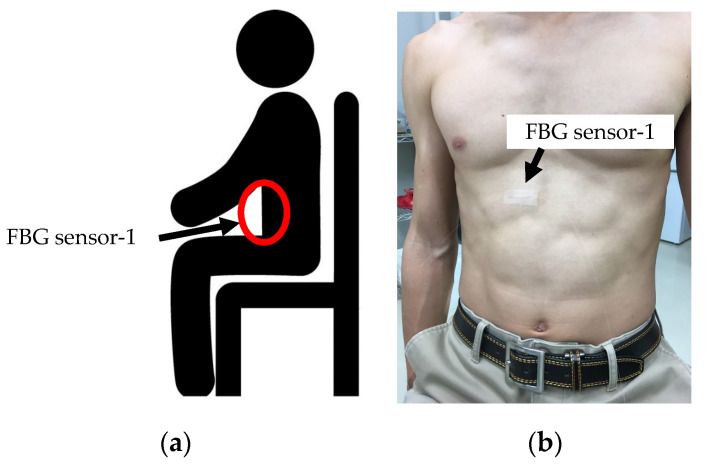
Subject’s posture and FBG sensor-installation position in measurement experiment of respiratory strain using FBG sensor (**a**): Subject’s posture, (**b**): FBG sensor installation position.

In our experiment, the FBG sensor waveforms during breathing and apnea were verified. We expected that the strain of the living body would differ greatly between breathing and apnea. As shown in Figure 4a, the subject’s measurement posture was sitting. This helped prevent strain caused by body movement. As shown in Figure 4b, the FBG sensor was installed on the abdomen. The subject repeated breathing for 30 s, and they then stopped breathing for 30 s. The time for one respiration was 6 s (inspiration: 3 s, expiration: 3 s), breathing was performed five times in 30 s. In these 60 s, the FBG sensor measured the strain from the living body. 

**Figure 5:**
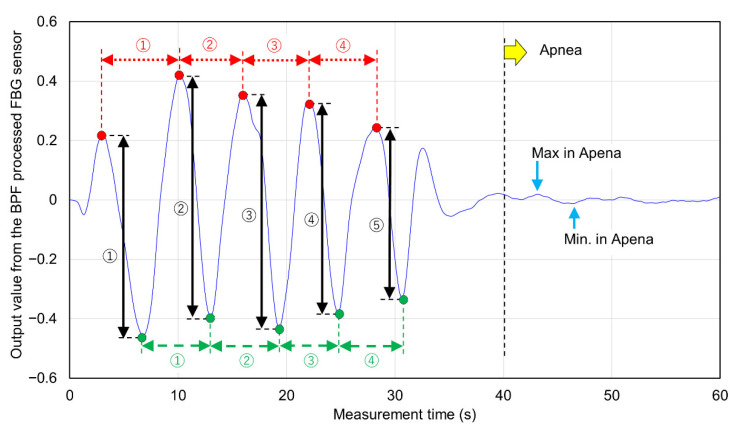
Analysis method of signals measured by FBG sensor.

The measured signal waveform was analyzed as shown in Figure 5. Figure 5 shows an example of the signal waveform measured during 5 rounds of breathing. Five top peaks of the measured signal waveform were detected (red plots in Figure 5), and the average value was calculated as the “average top peak value”. The breathing time for 4 breaths was calculated from the measurement time interval of the top peak (red-dotted arrows in Figure 5). Similarly, five bottom peaks of the measurement signal were detected (green plots in Figure 5), and the average value was calculated as the average bottom peak value. The time interval of the bottom peak was calculated (green-dashed arrows in Figure 5) as well as the top peak. The sum of the absolute values on the vertical axis from the top peak to the bottom peak detected was detected as the longest amplitude of one breath (black arrow in Figure 5). The longest amplitude during breathing was calculated from the average value of the five longest amplitudes. The reaction strain due to respiration was detected between 30 and 40 s after the end of respiration, the signal after 40 s was used as the signal measured in the apnea state. Of the signals after 40 s, the maximum output value on the vertical axis was detected as “Max in Apnea” and the minimum output value was detected as “Min. In Apnea” (sky blue arrow in Figure 5). The total of these absolute values was calculated as the longest amplitude in apnea. Furthermore, as the rate of change of the longest amplitude during respiration and apnea, the ratio between the longest amplitude value during respiration and the longest amplitude during apnea was calculated. The number of experiments was 5. In that experiment, the vertical direction of the FBG sensor signal due to respiration was verified. 

### 2.4. Measurement Experiment of Propagation Range of Respiratory Strain to each Living Body Part

In this experiment, two FBG sensors were installed on the living body. The subjects were three men in their twenties, measured in a sitting position. The subjects breathed for 30 s using a 6 s cycle. Five parts were selected for measuring the range of respiratory strain propagation: abdomen, chest, shoulders, elbows, and wrists. The abdomen and chest are the trunk of the living body and are the places where the movement of the diaphragm and lungs due to breathing can be detected. The elbow and wrist are the parts where the pulsating points of the arteries are located, and the strain due to the contraction of the heart can be detected. If respiratory strain propagates to these parts, it may be possible to measure cardiovascular and respiratory information at the same time by installing a single FBG sensor. The shoulder is the part that corresponds to the end of the trunk. The elbows and wrists are located beyond the shoulder or elbow joints when viewed from the trunk side of the abdomen and chest. If respiratory strain is measured at the shoulders only and not at the elbows or wrists, the result is that respiratory strain cannot propagate beyond the joints. For the above reasons, 5 measurement parts were selected.

**Figure 6:**
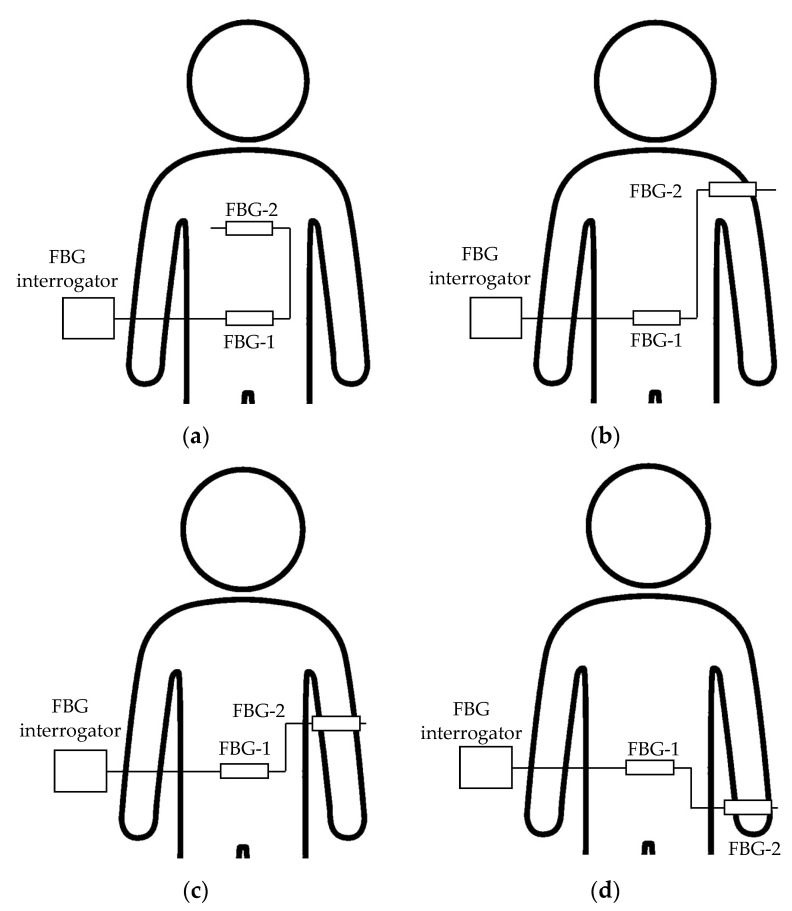
Image of the installation parts of each FBG sensor in the measurement experiment of the strain propagation range due to respiration to each living body part. (**a**): Abdomen and Chest, (**b**): Abdomen and Shoulder, (**c**): Abdomen and Elbow, (**d**): Abdomen and Wrist.

As shown in Figure 6a, FBG sensor-1 (*λ_Bragg_* = 1543 nm) was installed on the subject’s abdomen, and FBG sensor-2 (*λ_Bragg_* = 1561 nm) was installed on the chest. In this state, two FBG sensors were measured simultaneously. The signal measured by this FBG sensor-1 was used as a reference value for respiratory strain. Next, the FBG sensor-2 was installed on the shoulder and measured at the same time as FBG sensor-1 on the abdomen (Figure 6b). Then, the FBG sensor-2 was installed on the elbow or wrist (Figure 6c or Figure 6d), and the same measurement was performed; each part was measured 5 times.

Top peaks and bottom peaks were detected from the signal waveforms measured at each part, as with Figure 5. From these values, the average top peak value (Ave. Top peak), average bottom peak value (Ave. Bottom peak) and Longest amplitude value (Longest) were calculated. The ratio was calculated from the longest amplitude value of the abdomen measured at the same time as the longest amplitude value calculated for the chest, shoulders, elbows, and wrists (Longest amplitude value in abdomen/Longest amplitude in each part). When this ratio is smaller, it shows that the strain measured at each part is larger against the strain measured at the abdomen. When the ratio of the longest amplitude value to the abdomen measured at each part was smaller than the ratio of the longest amplitude value during breathing and apnea in Section 2.2, it was judged that respiratory strain could be measured. In this case, the time interval of the top peak of the signal measured at each part was detected, and this was defined as each respiratory time. The time interval of the top peak was also detected from the signal waveform of the abdomen measured at the same time. The measurement accuracy of the respiratory time calculation was verified from the correlation scatter plot of the respiratory time by each partial signal against the respiratory time by the abdomen signal.

On the other hand, if the ratio of the longest amplitude value to the abdomen measured at each part was larger than the ratio of the longest amplitude value during breathing and apnea, respiratory strain was not measured. In this case, this ratio indicates that only strains smaller than the amplitude value of strains unrelated to respiration measured during apnea are measured. Therefore, as a matter of course, the strain due to respiration cannot be measured by the FBG sensor. To prove this, the signal waveforms measured at each part were frequency-analyzed by Fourier transform. The subject was breathing in a 6 s cycle, and the breathing frequency was 0.167 Hz. Other frequencies were not respiratory strain. Therefore, if the frequency analysis shows a frequency other than 0.167 Hz, it means that the strain due to respiration could not be measured. From the above all results, the propagation range of strain due to respiration to each living body part was verified.

## 3. Results and Discussion

### 3.1. Simultaneous Measurement Result with Respiratory Temperature Sensor and FBG Sensors

**Figure 7:**
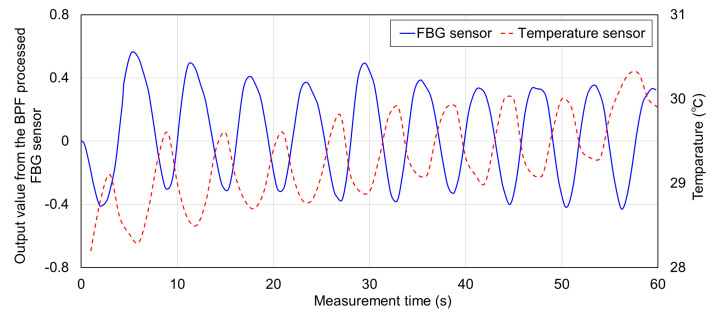
Signals measured by the FBG sensor and the temperature sensor.

Respiratory and FBG sensors installed in the oral cavity and on the abdomen were measured simultaneously. The signals measured by each sensor are shown in Figure 7. The time-series change of each is a vertically inverted waveform. During inspiration, the abdomen swelled, and the FBG sensor stretched, causing it to shift to the +side, and the respiratory sensor measured outside air that was cooler than the body temperature. Thus, the temperature decreased. Therefore, the FBG sensor and the respiratory sensor measured the inverted waveform.

The signal waveform measured by the FBG sensor was found to be detected at constant cycles. Therefore, these signals were predicted to be due to respiratory strain. The time interval of the top peak of the signal waveform of the FBG sensor and the time interval of the bottom peak of the corresponding temperature sensor were calculated as the breathing time of one cycle. Table 1 shows the average one cycle breathing time detected by the breathing temperature sensor and FBG sensor. The breathing time of one cycle by temperature sensor was detected all in 6 s, because the time resolution of this sensor was 1 s. On the other hand, the time resolution of the FBG sensor was 1000 Hz, and one cycle of respiration time was detected with high time resolution. The average respiration time in one experiment ranged from 6.010 to 6.128 s, and the average respiration time in all measurement data was 6.046 s. The standard deviation at this time was 0.327, and the respiration time could be measured with high accuracy, considering that the resolution of the temperature sensor was 1 s. Therefore, it was shown that the strain signal measured by the FBG sensor installed on the abdomen was almost the same as that of the respiratory strain. In the subsequent experiments, the FBG sensor signal installed on the abdomen was used as a reference respiratory strain signal.

### 3.2. FBG Sensor Measurement Signal Waveform Results during Breathing and Apnea

**Figure 8:**
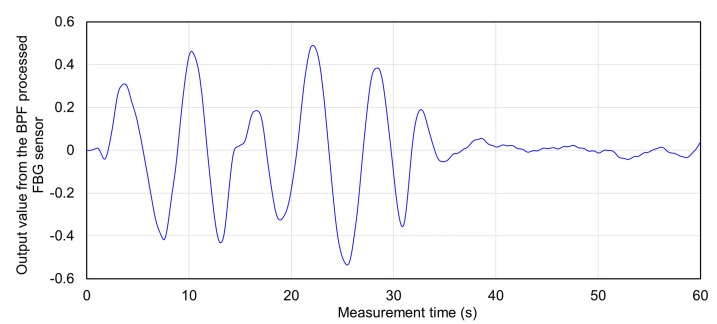
Measured FBG sensor signal during breathing and apnea in 3rd experiment (0–30 s: Breathing five times, 30–60 s: Apnea).

The strain signal during breathing and apnea was measured by the FBG sensor installed on the abdomen of the subject. The signals measured by the FBG sensor are shown in Figure 8. Because breathing was repeated five times from 0 to 30 s, a signal with a long amplitude was measured. The breathing was stopped after 30 s, a signal due to the recoil of respiratory strain with a shortened amplitude was detected, and after 40 s, the amplitude approached 0. The signal waveform measured by the FBG sensor differed greatly depending on the presence or absence of breathing. This signal waveform was analyzed in detail.

Table 2 shows the detection times and output values of the top and bottom peaks of the signal waveform of the FBG sensor measured when five experiments were performed. Five breaths were measured in one experiment, and the average peak time interval and output value for each breath are also shown. The average time interval between the top peak and the bottom peak was 5.750 to 6.287 s, which was almost 6 s. This time interval coincided with the time of the respiratory cycle set for the subject, and it was confirmed that this signal waveform corresponds to the respiratory strain. The output value of the top peak was 0.274 to 0.501, and the bottom peak was −0.532 to −0.373. The second experiment had the longest absolute values for both top and bottom peaks. It is considered that the cause of this was that the subject could not control the magnitude of respiration and that there was a difference in the pressure when the FBG sensor was installed in the living body. However, it was not a big problem because a large strain due to respiration could be measured in all experiments.

Table 3 shows the average output values of the top and bottom peaks in breathing and the maximum and minimum output values in apnea. This also shows the ratio of the longest amplitude values with and without breathing. The absolute value of the top and bottom peak output values during respiration was detected over 0.274. On the other hand, all output values during apnea were detected within ±0.05. Therefore, if the output value of the measurement signal of the FBG sensor was within ±0.05, it could be predicted that respiratory tension was not measured. The longest amplitude value during breathing was 0.632 to 1.033, and the average longest amplitude value was 0.774. The longest amplitude value during apnea ranged from 0.032 to 0.064, and the average longest amplitude value was 0.045. It was shown that the signal waveform of the FBG sensor differs significantly between respiration and apnea, because the longest amplitude values during respiration and apnea differ by more than 10 times. The ratio of the longest amplitude between breathing and apnea was from 11.1 to 22.2 times, an average ratio of 18.2 times. The largest value in this ratio, 22.2 times, was set as the standard. If a strain measurement signal is detected at a ratio of 22.2 times or less of the longest amplitude value of the FBG sensor measured at the same time in the abdomen, the measured strain may be a respiratory strain. Conversely, the strain measured at a ratio of 22.2 times or more is predicted to be a strain unrelated to respiration. From these results, the results of the range of the output value of the measurement signal of the FBG sensor and the ratio of the signal output measured against the abdomen was used as a judgment standard in the measurement experiment of the propagation range of respiratory strain to each living body part.

### 3.3. Measurement Result of Respiratory Strain Propagation Range

**Figure 9:**
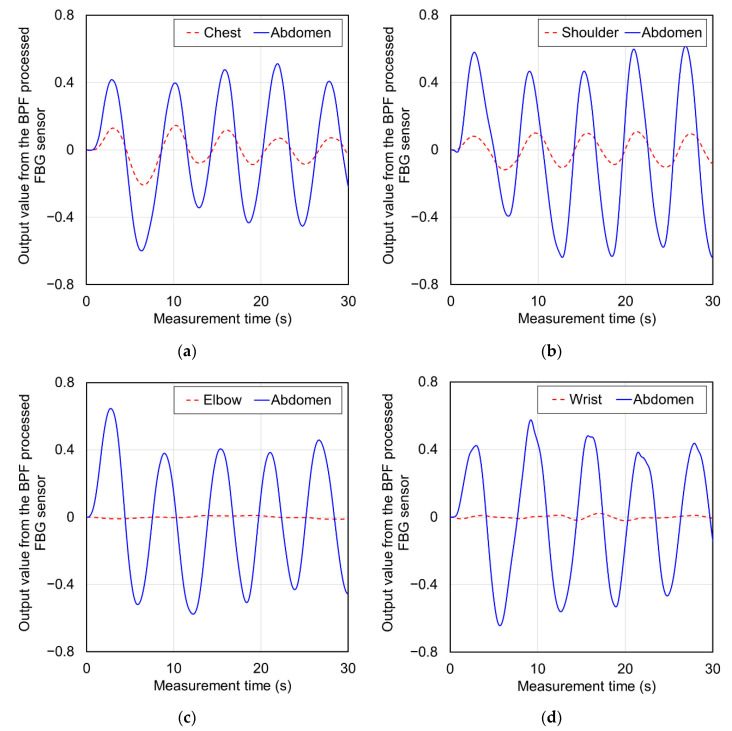
Measured signal waveforms on the chest, shoulder, elbow, wrist, and simultaneously measured on the abdomen. (**a**) Chest, (**b**) Shoulder, (**c**) Elbow, and (**d**) Wrist.

**Figure 10:**
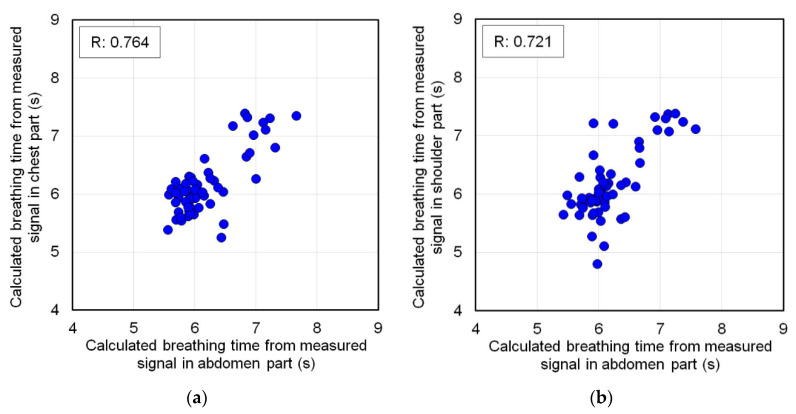
Scatterplot of breathing time from the signals measured at the chest and shoulder against the breathing time calculated from the abdominal signals. (**a**) Chest against abdomen, (**b**) Shoulder against abdomen.

The FBG sensor-1 installed on the abdomen of the subject and the FBG sensor-2 installed on each body part were simultaneously measured. Figure 9 shows the signal waveforms measured on the chest, shoulder, elbow, and wrist of subject-A and the those simultaneously measured on the abdomen. Additionally, Table 4 shows the average output and longest amplitude values of the top and bottom peaks of these measured signals. In the signal waveform of the FBG sensor installed in the abdomen, the absolute values of the output values of all the top and bottom peaks could be measured with a large strain exceeding ±0.4. The average peak interval time for these was about 6 s. Therefore, respiratory strain could be measured in the abdomen. In the signal waveforms measured at the chest and shoulder in Figure 9a,b, signals with the almost similar constant cycle as the abdomen were measured. The output values of these signals at the top and bottom peaks were over ±0.05, and the average peak interval time was about 6 s. Therefore, it was predicted that respiratory strain was measured in the chest and shoulder. The average longest amplitude values of the signals measured by each measuring points were 0.221 and 0.200. The average longest amplitude values of the signals in the abdomen measured at the same time were 0.898 and 1.105, respectively, and the ratio of the signal magnitude to the abdomen measured in the chest and shoulder was 4.1 and 5.5 times, respectively. Therefore, it was confirmed that the strain measured in the shoulder was smaller than the strain measured in the chest, because the average longest amplitude values of the signals in the abdomen measured at the same time were 0.898 and 1.105, respectively. In other words, respiratory strain can be measured in the abdomen, chest, and shoulders, and the magnitude of the measurable strain decreases as the distance from the abdomen increases. Figure 10 shows a scatterplot of breathing time from the signals measured at the chest and shoulder against the breathing time calculated from the abdominal signals simultaneously measured. The correlation coefficient was 0.764 in the chest and 0.721 in the shoulder, indicating a highly significant correlation. The reason why the correlation coefficient in the shoulder was lower than that in the chest was that the magnitude of the measurable strain was small. From these results, respiratory strain was measured in the chest and shoulder, and the respiratory time was calculated from the signal cycle.

**Figure 11:**
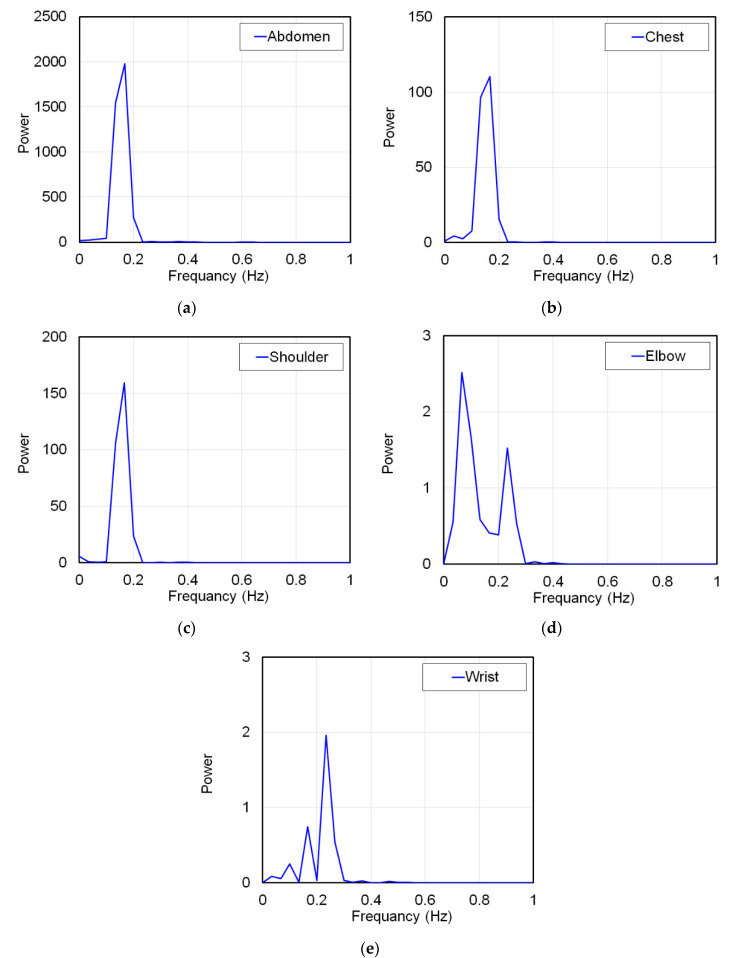
Frequency analysis results by Fourier transform of signals measured at each part of the living body, (**a**): abdomen, (**b**): chest, (**c**): shoulder, (**d**): elbow, (**e**): wrist.

On the other hand, in the elbows and wrists shown in Figure 9c,d, the constant cycle of respiratory strain as detected by the abdominal signal was not measured. From Table 4, the longest amplitude values of the elbow and wrist were 0.011 and 0.028, which were smaller than the reference output of respiratory strain of ±0.05. The ratio to the longest amplitude value of the abdomen measured at the same time was 87.4 in the elbow and 36.1 in the wrist, and the ratio in each part greatly exceeded the reference ratio for detecting respiratory strain, which was 22.2 times. From these results, it was predicted that respiratory strain could not be measured in the elbow and wrist. Figure 11 shows the results of frequency analysis by Fourier transform to confirm whether the strain due to respiration can be measured at each part. In Figure 11a–c, a peak of 0.167 Hz was confirmed in the abdomen, chest and shoulder. This frequency coincided with the respiratory time of 6 s, and respiratory strain could be measured at these points. The size of the power decreased in the order of abdomen, chest, and shoulder. The peak of 0.167 Hz could not be detected in the elbow and wrist, shown in Figure 11d,e, and these measurement signals did not include respiratory strain. In these results, the peaks at 0.067 or 0.233 Hz were the largest. These peaks were noise-induced frequencies because of their low power, and they were not related to respiratory strain.

From these results, the respiratory strain propagated from the abdomen to the shoulder, which is the end of the trunk. The magnitude of respiratory strain that could be detected in the trunk was the largest in the abdomen, and smaller in the chest and shoulder. At the shoulder, respiratory strain could not be detected with the fingertips, but the FBG sensor was able to detect the strain enough to calculate the respiratory time. However, respiratory strain did not propagate in the elbows and wrists located in the peripheral part off the trunk. It is possible that respiratory strain cannot propagate beyond the joints. Therefore, it was found that it is necessary to install it on the trunk in order to detect respiratory strain with an FBG sensor.

## 4. Conclusions

In this study, the strain on a living body caused by respiration was measured using an FBG sensor, and the measured signal waveform was verified. Additionally, respiratory strain was measured in the abdomen, chest, shoulder, elbow, and wrist, and the propagation range of this strain was verified. As a result, it was shown that the waveforms measured during breathing and apnea were significantly different. The respiratory cycle measured by the respiratory sensor and the peak interval time of the FBG sensor measurement signal waveform almost matched. Therefore, it was confirmed that the FBG sensor could accurately measure the respiratory strain. This strain signal was measured in the abdomen, chest, and shoulder, and the amplitude of the strain signal decreased with distance from the abdomen. This indicates that the respiratory strain propagated to the shoulder. Thus, the respiratory strain could be measured at any part of the trunk. On the other hand, respiratory strain was not measured in the elbows or wrists. Therefore, it was confirmed that the respiratory strain did not propagate to the peripheral parts of the body. From these results, we showed that respiratory strain propagates throughout the trunk, but not in the peripheral part.

Research on a sensor that can measure pulse rate and respiratory rate at the same time has been reported by Leal-Junior et al. [31]. We aim to develop a sensor system that can measure blood pressure and blood glucose level at the same time in addition to the simultaneous measurement of these two parameters. For that purpose, it is important that the strain due to breathing and the strain due to pulsation do not affect each other. In the signal that measures the pulsation strain, the signal due to respiratory strain is noise. Therefore, it affects the signal waveform to be analyzed, and the measurement accuracy for calculating blood pressure and blood glucose level decreases. In this paper, it was shown that respiratory strain did not propagate to the wrist, which measures pulsation strain. This is very important in our sensor system development. The developed FBG interrogator has two channels, it is possible to install two FBG sensors on the living body as shown in Figure 6d. The FBG sensor-1 is installed on the chest or abdomen as in this paper to measure respiratory strain. The respiratory rate can be calculated from this measurement signal. The FBG sensor-2 is installed at the pulsation point of the wrist and measures the pulsation strain. The pulse rate can be calculated from the average peak interval time of this measurement signal. A calibration curve has already been constructed focusing on the lengths of the top and bottom peaks (vertical axis direction) of the measurement signal, and blood pressure can be calculated from this calibration curve. The blood glucose level is also calculated from the calibration curve constructed by focusing on the measurement signal waveform. Pulse rate, blood pressure and blood glucose level can be calculated from one and the same signal, only the calculation method is different. As described above, the strain due to respiration and pulsation is measured by the two channel FBG interrogator, and multiple vital sign parameters, such as respiratory rate, pulse rate, blood pressure and blood glucose level can be calculated simultaneously from these signals.

Currently, many vital sign sensors are sold, but surprisingly few sensors can measure pulse rate and respiratory rate simultaneously. Electronic blood pressure monitors can measure pulse rate at the same time, but few can measure respiratory rate. However, in clinical settings, multiple sensors are required to simultaneously measure pulse rate, blood pressure, and respiratory rate. FBG sensors have the potential to overcome these problems. A wearable FBG interrogator was developed and can now be used as a wearable multi-vital sign sensor [30]. We expect this report to be an important step towards realizing the development of these sensors.

## Figures and Tables

**Table 1:** Average one-cycle respiratory time measured by the FBG sensor.

	Temp. Sensor	FBG Sensor	
	Ave. (s)	Ave. (s)	S.D.
1st	6	6.017	0.300
2nd	6	6.026	0.348
3rd	6	6.128	0.469
4th	6	6.010	0.191
Ave.	6	6.046	0.327

**Table 2:** Detection time and output value of top and bottom peaks measured by FBG sensor.

**Measurement Times**		**Breathing**					**Average**
**1st Time**		**1**	**2**	**3**	**4**	**5**	
**Top peak**	Time (s)	2.035	8.389	14.565	20.822	26.249	6.054
	Output	0.252	0.184	0.553	0.324	0.380	0.339
**Bottom peak**	Time (s)	5.959	12.368	17.868	24.180	29.637	5.920
	Output	−0.422	−0.265	−0.355	−0.319	−0.506	−0.373
**2nd time**		**1**	**2**	**3**	**4**	**5**	**----**
**Top peak**	Time (s)	3.523	10.687	16.340	22.560	28.277	6.189
	Output	0.805	0.241	0.371	0.524	0.566	0.501
**Bottom peak**	Time (s)	7.692	13.510	19.463	25.182	30.691	5.750
	Output	−0.574	−0.543	−0.676	−0.530	−0.336	−0.532
**3rd time**		**1**	**2**	**3**	**4**	**5**	**----**
**Top peak**	Time (s)	3.676	10.247	16.599	22.112	28.405	6.182
	Output	0.310	0.462	0.186	0.489	0.385	0.366
**Bottom peak**	Time (s)	7.537	13.077	18.884	25.459	30.858	5.830
	Output	−0.418	−0.431	−0.325	−0.535	−0.356	−0.413
**4th time**		**1**	**2**	**3**	**4**	**5**	**----**
**Top peak**	Time (s)	4.328	9.850	15.938	22.042	27.839	5.878
	Output	0.258	0.195	0.438	0.377	0.099	0.274
**Bottom peak**	Time (s)	6.807	12.941	18.784	24.907	31.150	6.086
	Output	−0.428	−0.262	−0.356	−0.421	−0.326	−0.359
**5th time**		**1**	**2**	**3**	**4**	**5**	**----**
**Top peak**	Time (s)	3.055	10.144	15.966	21.928	28.202	6.287
	Output	0.224	0.421	0.359	0.327	0.248	0.316
**Bottom peak**	Time (s)	6.763	12.977	19.302	24.743	30.640	5.969
	Output	−0.457	−0.392	−0.430	−0.379	−0.329	−0.397

**Table 3:** Average output values of top and bottom peaks in breathing and the maximum and minimum output values in apnea. (Ave.: Average, Max.: Maximum, Min.: Minimum)

	Ave. Output Value in Breathing			Ave. Output Value in Apnea			Ratio (A/B)
	Top Peak	Bottom Peak	Longest (A)	Max.	Min.	Longest (B)	
1st	0.339	−0.373	0.712	0.040	−0.024	0.064	11.1
2nd	0.501	−0.532	1.033	0.002	−0.050	0.052	19.8
3rd	0.366	−0.413	0.780	0.025	−0.012	0.038	20.7
4th	0.274	−0.359	0.632	0.015	−0.022	0.037	17.0
5th	0.316	−0.397	0.713	0.019	−0.013	0.032	22.2
Ave.	0.359	−0.415	0.774	0.020	−0.024	0.045	18.2

**Table 4:** Top peak, bottom peak, and the longest amplitude of the measurement signals at the chest, shoulders, elbows, and wrists, measured simultaneously with the abdomen.

	Each Part			Abdomen			Ratio (A/B)
	Ave. Top Peak	Ave. Bottom Peak	Longest (B)	Ave. Top Peak	Ave. Bottom Peak	Longest (A)	
Chest	0.107	−0.114	0.221	0.442	−0.456	0.898	4.1
Shoulder	0.097	−0.103	0.200	0.545	−0.560	1.105	5.5
Elbow	0.005	−0.006	0.011	0.455	−0.506	0.961	87.4
Wrist	0.014	−0.014	0.028	0.460	−0.550	1.010	36.1
